# SPA4 peptide attenuates the accumulation of selected damage-associated molecules and pulmonary cellularity during acute *Pseudomonas aeruginosa* infection in mice

**DOI:** 10.1371/journal.pone.0362301

**Published:** 2026-11-11

**Authors:** Shanjana Awasthi, Bhupinder Singh, Jun Xie

**Affiliations:** Department of Pharmaceutical Sciences, University of Oklahoma Health Campus Oklahoma City, Oklahoma, United States of America; Lady Hardinge Medical College, INDIA

## Abstract

Activation of Toll-like receptor 4 (TLR4) and accumulation of damage-associated molecular patterns (DAMPs) are hallmarks of infection and inflammation. The SPA4 peptide (amino acid sequence: GDFRYSDGTPVNYTNWYRGE) modulates TLR4-dependent host defense against *Pseudomonas aeruginosa*. In this study, we investigated the expression of TLR4, major DAMPs, and cellularity in the lungs of *P. aeruginosa*-infected mice treated with SPA4 peptide or vehicle. The expression of TLR4 and levels of DAMPs were analyzed by immunoblotting and enzyme-linked immunosorbent assay, respectively. Cellularity was determined in hematoxylin and eosin-stained lung tissue sections by microscopy. Our results demonstrated that the expression of TLR4 and DAMPs, as well as tissue cellularity, were increased in the lungs of infected mice. While *P. aeruginosa*-induced TLR4 expression was not affected, the levels of DAMPs were reduced in mice therapeutically treated with SPA4 peptide (fibrinogen: 8,730 ng; fibronectin: 17,925 ng; High Mobility Group Box-1 [HMGB-1]: 11,758 ng; and hyaluronan: 34,157 ng per g lung weight) compared with vehicle-treated mice (fibrinogen: 13,178 ng; fibronectin: 22,348 ng; HMGB-1: 15,896 ng; and hyaluronan: 53,644 ng per g lung weight). Correspondingly, reduced cellularity was noted in the lungs of SPA4 peptide-treated mice. These results advance our understanding of the SPA4 peptide activity against infectious stimuli.

## Introduction

Uncontrolled lung infection and inflammation can cause lung injury and acute respiratory distress syndrome resulting in unwanted severe health outcomes. These conditions are often associated with unresolved inflammation accompanied by the accumulation of stress ligands or damage-associated molecular patterns (DAMPs). While pathogen-associated molecular patterns (PAMPs) are molecules derived from pathogens, DAMPs are a heterogenous group of molecules produced by damaged host cells [1].

DAMPs are released in large amounts in the lung by local cells as well as through their leakage from plasma, during lung infections and inflammation caused by direct pulmonary or systemic exposure to pathogens or non-infectious stimulants. Both PAMPs and DAMPs accumulate rapidly in lung tissue and in circulation. Tissue injury initiates the production and accumulation of DAMPs within a short period of time through enhanced *de novo* synthesis, translocation, and secretion mediated by increased activity of synthetic or modifying enzymes and reactive oxygen species [2–9]. Gram-negative bacterial lung infection caused by *Pseudomonas aeruginosa* also results in the release of lipopolysaccharide (LPS, a major PAMP) as well as DAMPs [10]. PAMPs and some DAMPs are recognized by the pattern-recognition receptors (PRRs) expressed by host immune and non-immune cells. One predominant class of PRRs is Toll-like receptors (TLRs), which are ubiquitously expressed by both immune and non-immune cells. Upon recognition of PAMPs or DAMPs, TLR is activated, inducing an innate immune response that includes inflammatory signaling. The priming step of TLR activation can further induce intracellular inflammasomes resulting in robust production of inflammatory cytokines, such as IL-1β. Persistent stimulation of TLRs orchestrates innate immunity and drives a sustained inflammatory response [11].

Among several TLRs known to recognize the PAMPs derived from different pathogens in a distinct manner, TLR4 recognizes LPS of Gram-negative bacteria as well as certain DAMPs. Although the molecular mechanism of TLR4 activation by DAMPs remains poorly defined, substantial insight has been gained regarding LPS sensing. Following binding of LPS to lipid-binding protein and CD14 and its transfer to an adaptor myeloid differentiation factor-2 (MD2) protein, TLR4 forms homodimeric TLR4-MD2 complex, engages intracellular co-receptors (myeloid differentiation primary response gene 88 [MYD88] and Toll/interleukin-1 receptor domain-containing adapter-inducing interferon-β [TRIF]), and activates downstream signaling pathways [12,13].

While TLR4 promotes recognition and uptake of Gram-negative bacterial pathogens, TLR4 signaling stimulates the expression of inflammatory cytokines, chemokines, and chemical mediators. However, a robust inflammatory response can enhance pathogen growth and proliferation and cause exaggerated inflammatory responses and tissue injury [11,14]. Injured or stressed cells during these processes of innate immune response release a variety of DAMPs, some of which, such as fibrinogen, fibronectin, High Mobility Group Box-1 (HMGB-1), and hyaluronan, are recognized by TLR4 [15].

Using TLR4-overexpressing cells, our results have demonstrated that the SPA4 peptide (amino acid sequence: GDFRYSDGTPVNYTNWYRGE) suppresses TLR4-dependent inflammatory responses, while promoting phagocytosis through modulation of TLR4 signaling against LPS and Gram-negative bacteria, including *Escherichia coli* and *P. aeruginosa* [16,17]. Modulation of TLR4 signaling by the SPA4 peptide involves suppression of LPS- and ATP-induced NLRP3 inflammasome and altered gene and protein expression of endoplasmic reticulum-associated and histone proteins [17,18]. Therapeutic treatment with the SPA4 peptide suppresses pathogen burden, inflammation, and tissue injury in *P. aeruginosa*- and LPS-induced lung infection and inflammation models [16,19–22]. Our results further demonstrated that SPA4 peptide treatment suppresses the expression of total and phosphorylated NF-κB p65, as well as phosphorylated p38 mitogen-activated protein kinase (MAPK) and p54 stress-activated protein kinase (SAPK)/c-Jun N-terminal kinase (JNK) in lung [16]. In addition to the presence of LPS, the most potent ligand of TLR4, the accumulation of DAMPs during infection may substantially influence the magnitude of the host response and its associated outcomes. Endogenous DAMPs are continuously released in response to infectious and inflammatory stimuli through mechanisms described as the “TLR4 radical cycle” or “danger chain reaction”, which promote persistent and chronic inflammation [23–26]. Based on our previous findings demonstrating that the TLR4-interacting SPA4 peptide exhibits both pro-phagocytic and anti-inflammatory activities, leading to enhanced pathogen clearance and reduced inflammation [16,17], we hypothesized that SPA4 peptide treatment may also modulate DAMP levels, thereby interrupting the TLR4-driven inflammatory cycle and promoting the resolution of inflammation. To date, the effects of SPA4 peptide treatment on the accumulation of DAMPs during acute bacterial lung infection have not been investigated. Therefore, in this study, we evaluated the steady-state expression levels of TLR4 and DAMPs, as well as total cellularity (cell counts per unit area), in the lung tissues of *P. aeruginosa*-challenged mice treated with SPA4 peptide. Our results demonstrate that SPA4 peptide treatment does not affect TLR4 expression levels but significantly reduces the levels of fibrinogen, fibronectin, HMGB-1, and hyaluronan, as well as cellularity, in the lungs of mice challenged with live *P. aeruginosa*.

The findings presented here advance our understanding of SPA4 peptide activity in an acute bacterial lung infection model and provide a foundation for future studies of SPA4 peptide and other multifunctional peptides currently under investigation by several research groups.

## Methods

### SPA4 peptide

The SPA4 peptide (amino acid sequence: GDFRYSDGTPVNYTNWYRGE; Genscript, Piscataway, NJ) was included in this study. The purity of the SPA4 peptide was confirmed by mass spectroscopy and high-performance liquid chromatography (HPLC). The SPA4 peptide was reconstituted in endotoxin-free water and stored frozen at −20°C. Heat-inactivated SPA4 peptide was prepared by heating the SPA4 peptide solution at 99°C for 4 h.

### Pseudomonas aeruginosa

*P. aeruginosa* PAO1 bacterial cultures were maintained in tryptic soy broth or agar medium [27]. Gram staining was performed using a kit (BD Biosciences, Sparks, MD). Bacterial characteristics were confirmed by catalase and oxidase enzyme analyses (BD Biosciences, San Jose, CA) according to the manufacturer’s instructions. Briefly, bacterial colonies were mixed with a few drops of catalase test reagent on a glass slide; immediate bubbling confirmed a catalase-positive reaction. For the oxidase test, colonies were smeared onto filter paper moistened with oxidase test reagent; development of a blue color confirmed oxidase positivity.

After confirmation of Gram-negative staining and biochemical characteristics, a single bacterial colony was inoculated into 5 ml of trypticase soy broth and incubated overnight at 37 °C in a rotatory incubator. The following day, 0.2 ml of overnight culture was added to 20 ml fresh sterile trypticase soy broth medium and incubated for 4 h. Absorbance was measured at 600 nm wavelength to confirm log-phase growth based on a predetermined growth curve. The required volume of bacterial culture was pelleted by centrifugation (3,220 x g for 20 min at 4 °C), washed, and re-suspended in 0.15M sterile NaCl. Absorbance was again measured at 600 nm wavelength against sterile 0.15M NaCl, and bacterial suspensions were diluted to obtain ~1 x 10^7^ colony forming units (CFU) in 25 µl. Live CFU counts were confirmed by plating diluted inoculum on tryptic soy agar plates and enumerating colonies. Live bacterial counts ranged from 1.33 x 10^7^ to 1.38 x 10^7^ CFU per 25 µl for intratracheal challenge per mouse.

### Mice

C57BL/6J female mice (5–6 weeks old; Jackson Laboratory, Bar Harbor, ME) were used in this study. All mice were acclimatized for at least one week prior to experimentation and were randomly assigned to study groups. Mice were given food and water *ad libitum*. The study duration was 5 h, during which no animal died or met the criteria for humane endpoints. All animals were monitored at 0–3 h and again at 4–5 h during the study. Mice were anesthetized using isoflurane followed by ketamine/xylazine anesthesia. Anesthetized mice were euthanized by cervical dislocation. All animal protocols were approved by the University of Oklahoma Health Campus (OUHSC) Institutional Animal Care and Use Committee (IACUC; Protocol numbers 14–054-HI, 12–105-HI, and 17–081-CHR). All research personnel were trained in animal care and handling. Experimental procedures were performed in accordance with approved guidelines and regulations and were compliant with applicable animal welfare requirements.

### Therapeutic administration of SPA4 peptide in mice challenged with *P. aeruginosa*

Anesthetized mice were positioned on the intubation platform and intratracheally challenged with ~1 x 10^7^ CFU of bacteria (per OD_600_) using a gel-loading pipette tip and a laryngoscope (Penn-Century, Wyndmoor, PA), as described previously [16]. One hour after bacterial challenge, mice were treated intratracheally with 50 μg of SPA4 peptide, an equivalent amount of heat-inactivated SPA4 peptide, or an equivalent volume of endotoxin-free water (vehicle). Mice were sacrificed 5 h after bacterial challenge. Unchallenged, untreated mice were included as additional controls.

Lung tissue pieces were aseptically collected at necropsy and were either immediately frozen in liquid nitrogen or fixed in 10% formalin. Tissue samples were collected from unspecified lung lobes. Frozen lung tissue pieces were thawed, weighed, and homogenized in ice-cold lysis buffer containing mild detergent and protease inhibitors (100 mg tissue per ml lysis buffer). Homogenates were centrifuged at 367 x g for 10 min using Beckman Coulter Microfuge 18 at 4 °C. Supernatants were collected, and the homogenization step was repeated with the pellet. Supernatants were pooled, aliquoted, and stored at −80 °C. Total protein concentrations in diluted lung tissue homogenates were measured using the Pierce Bicinchoninic acid (BCA) assay kit according to the manufacturer’s instructions (Thermo Fisher Scientific, Waltham, MA). Formalin-fixed lung tissues were processed, sectioned, and stained with hematoxylin and eosin (H & E).

### TLR4 expression by western immunoblotting

Forty micrograms of lung tissue homogenate protein were separated on 4–20% Tris-glycine gel (Invitrogen, Carlsbad, CA) under partially reducing conditions (in the presence of β-mercaptoethanol reducing agent, without heating). Proteins were immunoblotted with a TLR4-specific antibody (2 μg/ml, catalog number 13867, Abcam, Waltham, MA) followed by stripping and re-immunoblotting with β-actin specific antibody (1:5,000 dilution, catalog number 4967, Cell Signaling Technology, Danvers, MA) as described previously [19]. The secondary antibody was obtained from Cell Signaling Technology, Danvers, MA (Catalog number 7074). Blocking and detection reagents were obtained from Thermo Fisher Scientific. Densitometric values of TLR4 immunoreactive bands were normalized to β-actin.

### Measurement of major DAMPs (HMGB-1, fibronectin, fibrinogen, and hyaluronan) by immunoassays

Commercially available ELISA kits were used to measure levels of total fibrinogen (Innovative Research, Novi, MI), fibronectin (Abcam), HMGB-1 (Novateinbio, Woburn, MA), and hyaluronan (RnD Systems, Minneapolis, MN) in 1:50 or 1:100 diluted lung tissue homogenates, per the manufacturers’ instructions. These dilutions were used to allow measurement of the highest analyte concentrations within the detection limits of the respective ELISA kits. Measured levels of DAMPs were normalized to lung wet weight (g).

### Quantitation of cellularity in lung tissues

We previously reported that vehicle treatment resulted in marked hypercellularity in lungs, mainly consisting of neutrophilic leukocytes, against *P. aeruginosa* challenge, whereas lungs of SPA4 peptide-treated mice were only mildly cellular [16]. In this study, tissue cellularity was quantified in H & E-stained lung tissue sections from *P. aeruginosa*-challenged mice treated with SPA4 peptide or controls. Five to nine images were collected from randomly selected regions of one lung tissue section from each mouse, excluding areas containing vasculature. Images were acquired using a fixed region with a side length of 680 px. Cells with identifiable nuclei were counted within each frame twice independently on separate occasions in blinded fashion using the STEPanizer program [28]. Results on tissue cellularity or total cell counts per unit area, presented here should be viewed in conjunction with our previously published results on inflammatory cell infiltration and tissue damage [16].

### Statistical analysis

Results were analyzed using Analysis of variance (ANOVA) with Tukey’s post hoc test for statistical significance (GraphPad prism, San Diego, CA). A *p* < 0.05 was considered statistically significant unless otherwise indicated. The JMP Statistical Discovery program (Cary, NC) was used to generate distribution and quantile box plots for individual readings of lung tissue cellularity among study groups.

## Results

### No effect of SPA4 peptide on *P. aeruginosa* PAO1-stimulated expression of TLR4

Two immunoreactive bands of TLR4 protein were detected in lung homogenates run under partially reducing conditions (Fig 1). The densitometric units of immunoreactive bands 1 (high molecular weight) and 2 (low molecular weight) of TLR4 were normalized to those of a single immunoreactive band of β-actin. As expected, increased expression levels of TLR4 protein were observed in lung tissues of *P. aeruginosa*-challenged, vehicle-treated mice (mean densitometric ratios for immunoreactive bands 1 and 2: 0.63 and 0.61, respectively) compared with unchallenged, untreated mice (0.44 and 0.34, respectively).

**Fig 1:**
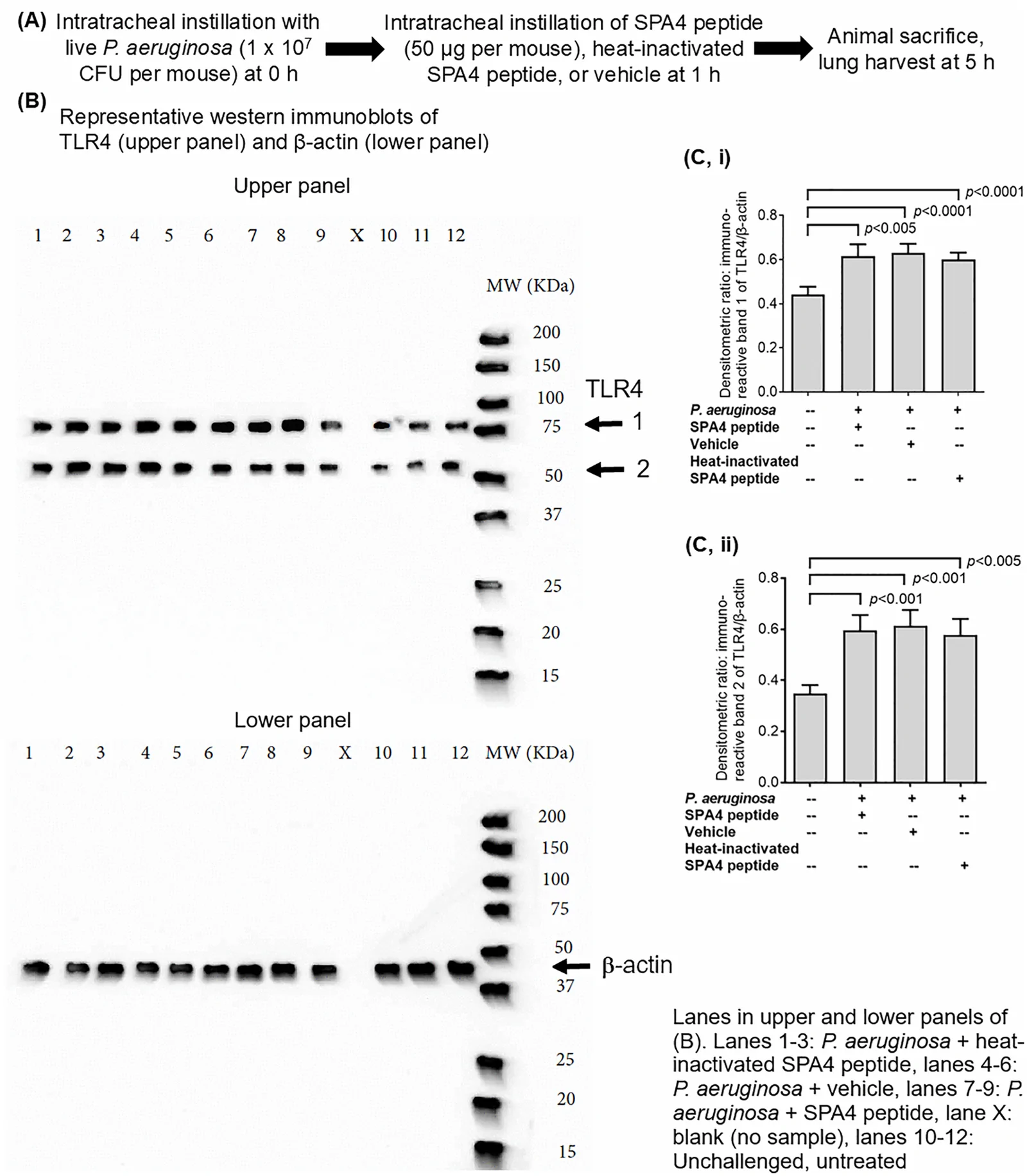
Expression of TLR4 in lung tissue. Time schedule of *P. aeruginosa* challenge, treatment with SPA4 peptide, and organ harvest are shown in (A). The TLR4 expression was studied in lung tissue proteins by western immunoblotting, The lung tissue proteins were separated by gel electrophoresis under partially reducing conditions prior to immunoblotting for TLR4 followed by stripping of membrane and re-immunoblotting for β-actin. Immunoblotting was performed using tissue pieces from n = 11 *P. aeruginosa* + SPA4 peptide-treated mice, n = 12 *P. aeruginosa* + vehicle-treated mice, n = 10 *P. aeruginosa* + heat-inactivated SPA4 peptide-treated mice, included across two independent experiments. Tissue pieces from four unchallenged, untreated mice were included as control. Three samples from each group were included in four immunoblots run on separate occasions. Results presented in (B) and (C) are representative of a total of n = 12 immunoblotting assessments per group. The densitometric ratios for immunoreactive bands of TLR4 are shown in (C, i and ii). Statistical significance was studied by ANOVA with Tukey’s post-hoc test.

The expression of TLR4 remained unaltered in lungs of *P. aeruginosa*-challenged mice treated with SPA4 peptide (0.61 and 0.59) or heat-inactivated SPA4 peptide (0.6 and 0.58). The expression of TLR4 was significantly increased in lungs of *P. aeruginosa*-challenged, vehicle-treated (*p* < 0.0001, *p* < 0.001), SPA4 peptide-treated (*p* < 0.005, *p* < 0.001) and heat-inactivated SPA4 peptide-treated mice (*p* < 0.0001, *p* < 0.005) compared with unchallenged, untreated mice (Fig 1).

### Suppression of DAMPs in *P. aeruginosa* PAO1-infected lungs after SPA4 peptide treatment

The sensitivities of the ELISA systems were 0.78 ng/ml (fibrinogen), 4.7 ng/ml (fibronectin), 3.75 ng/ml (HMGB-1), and 1.25 ng/ml (hyaluronan). The ELISA kits for fibrinogen and hyaluronan detected total mouse fibrinogen and ≥35 kDa hyaluronan among different molecular species of hyaluronan, respectively.

Significantly increased levels of fibrinogen (13,178 ng/g lung; Fig 2A), fibronectin (22,348 ng/g lung; Fig 2B), HMGB-1 (15,896 ng/g lung; Fig 2C), and hyaluronan (53,644 ng/g lung; Fig 2D) were observed in lungs of *P. aeruginosa*-challenged, vehicle-treated mice compared with unchallenged, untreated mice (fibrinogen: 4,150 ng/g; fibronectin: 11,672 ng/g; HMGB-1: 5,205 ng/g; hyaluronan: 19,206 ng/g lung).

**Fig 2:**
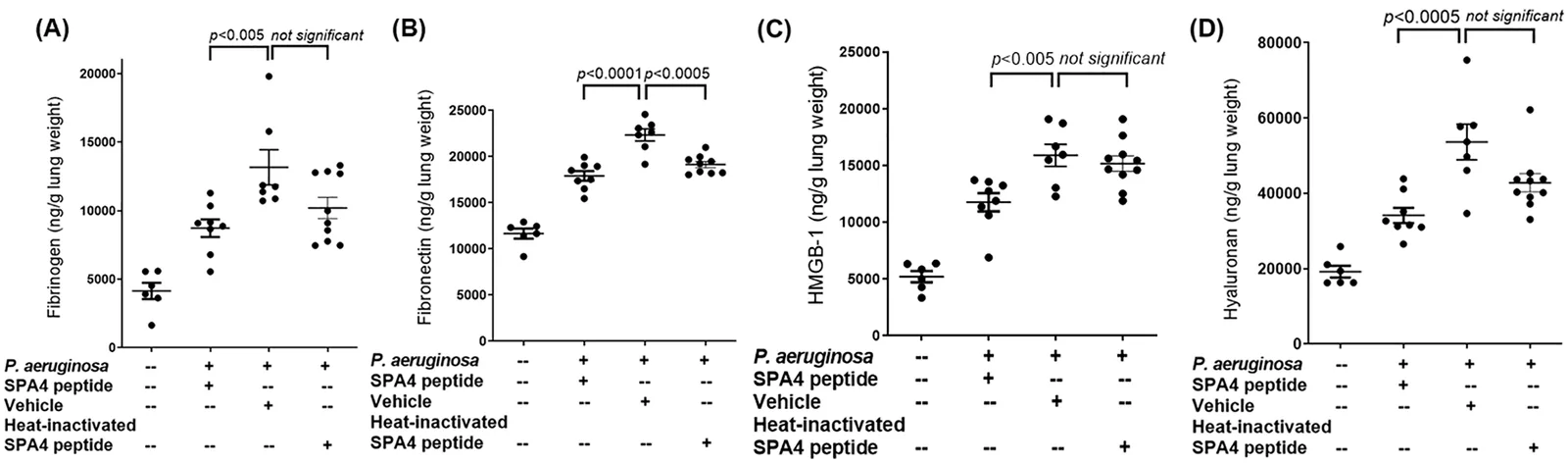
Measured amounts of major DAMPs in lung tissue homogenates. The levels of fibrinogen (A), fibronectin (B), HMGB-1 (C) and hyaluronan (D) were measured by commercially available ELISA kits. Results are from n = 6-10 mice per group included in two separate experiments. Each lung tissue homogenate sample was evaluated in triplicate wells and measured optical density values were averaged for quantification of DAMP levels and normalized with g lung tissue weight. Statistical significance was determined by using ANOVA with Tukey’s post-hoc test.

Treatment with SPA4 peptide significantly suppressed the levels of all measured DAMPs (fibrinogen: 8,730 ng/g lung, *p* < 0.005; fibronectin: 17,925 ng/g lung, *p* < 0.0001; HMGB-1: 11,758 ng/g lung, *p* < 0.005; and hyaluronan: 34,157 ng/g lung, *p* < 0.0005) in *P. aeruginosa*-challenged mice. Treatment with heat-inactivated SPA4 peptide only affected the accumulation of fibronectin (*p* < 0.0005) but did not affect the *P. aeruginosa*-induced levels of fibrinogen, hyaluronan, and HMGB-1 (statistically not significant) compared with vehicle-treated mice.

### Reduced cellularity in lung tissues of SPA4 peptide-treated mice

As expected, the cellularity was increased in lung tissues of *P. aeruginosa*-challenged, vehicle-treated mice compared with unchallenged, untreated mice. Significantly less tissue cellularity was observed in SPA4 peptide treatment group (Fig 3A).

**Fig 3:**
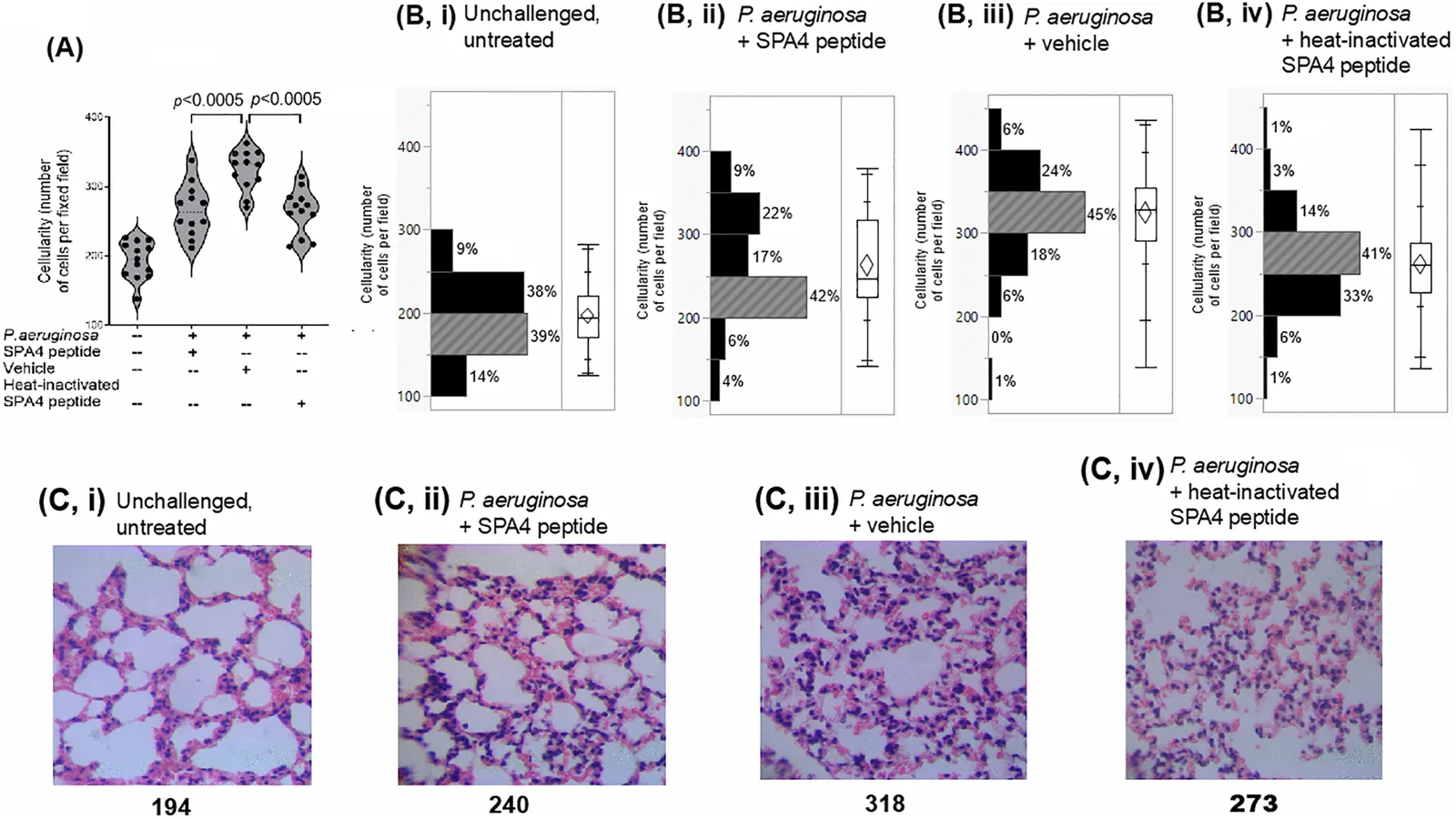
Cellularity in H & E-stained lung tissue sections. A lung tissue section was harvested from each mouse in study groups of *P. aeruginosa*-challenge and SPA4 peptide-, vehicle- or heat-inactivated SPA4 peptide-treatment. The lung tissue sections from unchallenged, untreated mice were included as controls. Results are from n = 12 mice per group included in two separate experiments, or from n = 13 unchallenged, untreated mice on seven separate occasions. The cells were counted per fixed field within five to nine collected images from unspecified regions of one randomly collected tissue per mouse. Each data point in (A) denotes an average of these cell counts per mouse in a study group; statistical significance on these data points was determined by using ANOVA with Tukey’s post-hoc test (A). The distribution plots of individual cell count per fixed field in all the images from mice in specific study groups are shown in (B, i-iv). The shaded area denotes the maximum percentage of images demonstrating cells count in each subgroup (cell counts per field 100-150, 150-200, 200-250, 250-300, 300-350, 350-400, 400-450). The quantile box plot for each group is shown within the figure (B, i-iv). Representative photomicrographs of lungs of mice in each study group are included in (C, i-iv), and the number of cells per fixed field is indicated at the bottom of each image.

The distribution plot analysis of individually determined total cell counts per unit area (tissue cellularity) from five to nine images per lung section of each mouse revealed within-group variability and distinct differences among the experimental groups: the maximum (39%) of unchallenged, untreated mice exhibited 150–200 cells (range 124–283; median = 194), whereas 45% of *P. aeruginosa*-challenged, vehicle-treated mice exhibited 300–350 cells (range 138–437; median = 329) cells. In comparison, 41% of *P. aeruginosa*-challenged, heat-inactivated SPA4 peptide-treated mice exhibited 250–300 cells (range 136–424; median = 260), and 42% of *P. aeruginosa*-challenged, SPA4 peptide-treated mice exhibited 200–250 cells (range 141–380; median = 246) within the fixed region across multiple images per lung (Fig 3B, i-iv).

## Discussion

Our previously published results demonstrated that the SPA4 peptide treatment increases phagocytic uptake and lysis of *P. aeruginosa* and *E. coli* while reducing inflammatory cytokine responses [16,17]. In TLR4-overexpressing cells, SPA4 peptide promotes TLR4-mediated phagocytic uptake and localization of bacteria in phagolysosomes while suppressing TLR4-dependent inflammatory response against P. aeruginosa and E. coli [16,17]. Simultaneous pro-phagocytic and anti-inflammatory activities of SPA4 peptide result in reduced bacterial burden, inflammation, and lung injury in a mouse model of *P. aeruginosa* lung infection [16]. Additionally, SPA4 peptide treatment altered the subcellular distribution of TLR4 in time-dependent manner in association with co-localization with bacteria without affecting overall receptor expression [17], suggesting that modulation of TLR4 trafficking likely contributes to its biological effects.

Consistent with these findings, the results presented here demonstrate that *P. aeruginosa*-induced TLR4 expression is not altered in lungs after SPA4 peptide treatment. Two immunoreactive bands of TLR4 were detected in murine lung homogenates (Fig 1), similar to those previously reported in baboon lungs [29,30]. These findings suggest likelihood of the presence of both soluble (low molecular weight immunoreactive band 2 without transmembrane and cytoplasmic domains) [31] and membrane-associated (high molecular weight immunoreactive band 1) forms of TLR4. These assumptions will be evaluated comprehensively in future investigations. Expression patterns of both bands were consistent across treatment groups. The levels of both bands were lowest in unchallenged, untreated mice, with particularly reduced levels of the soluble TLR4 form. Increased levels of soluble TLR4 during bacterial infection have been reported previously [32], and elevated soluble TLR4 has been suggested as a biomarker for urinary tract infection and pyelonephritis [33].

The LPS and LPS-expressing Gram-negative bacteria are well known to stimulate TLR4 signaling and induce inflammation and tissue injury. As tissue injury progresses, endogenous stress ligands or DAMPs are released into the extracellular milieu, some of which act as “feed forward” stimulators of TLR4 signaling. While specific regions of DAMPs– fibrinogen cleavage products, the EDA of fibronectin, Box B domain of HMGB-1, and low-molecular-weight fragments of hyaluronan have been implicated in binding to TLR4 and stimulation of inflammatory signaling; the immunoassays used in this study measured total fibrinogen, fibronectin, HMGB-1, and ≥35 KDa hyaluronan, limiting the ability to define specific regions responsible for binding to TLR4 and pro-inflammatory effects.

Among the DAMPs examined, fibrinogen and fibronectin are particularly relevant because they are implicated in both bacterial adherence and TLR4 activation. Fibrinogen promotes bacterial adherence to host cells and its expression increases as part of the systemic acute-phase response [34]. Several studies have demonstrated that lung epithelial cells express fibrinogen in response to infectious and inflammatory stimuli [35,36]. At sites of tissue injury, fibrinogen is converted to fibrin by thrombin, releasing fibrinopeptides that subsequently polymerize to form a fibrin matrix. This matrix is then degraded by plasmin into fragments. In addition, other host-derived and pathogen-derived proteases can degrade fibrin, generating a diverse array of fibrinogen cleaved products [37]. *P. aeruginosa* strains express elastase and proteinase enzymes capable of cleaving fibrinogen [38,39]. Some fibrinogen-derived cleaved products as well as modified citrullinated fibrinogen, can stimulate inflammatory response through TLR4 activation [40]. Although the specific fibrinogen cleaved products that act as TLR4 ligands have not yet been fully identified, elevated total plasma or serum fibrinogen levels have been reported in patients with non-cystic fibrosis bronchiectasis and airway *P. aeruginosa* infection [41], as well as in a rat model of *P. aeruginosa* infection [42]. Therefore, the elevated tissue fibrinogen levels observed in our study (Fig 2) indicate a potential role for fibrinogen in amplifying inflammation during infection, while the reduction in fibrinogen following SPA4 peptide treatment substantiates its anti-inflammatory effects.

Fibronectin, a ubiquitously expressed extracellular matrix glycoprotein, plays critical roles in multiple biological processes and is upregulated during infection and inflammation [43]. Several pathogens, including *P. aeruginosa*, exploit fibronectin for adherence to host cells, thereby facilitating bacterial colonization and infection [44,45]. Consequently, the suppression of both fibrinogen as well as fibronectin by SPA4 peptide treatment may be associated with the reduced bacterial adherence, colonization, and burden. Two major forms of fibronectin have been described: plasma fibronectin, a soluble dimer, secreted by hepatocytes that lacks the extra domain A (EDA) and EDB sequences, and cellular fibronectin, a multimeric extracellular matrix form, that contains EDA and EDB domains [46,47]. The EDA domain of cellular fibronectin can bind TLR4 and promote inflammatory signaling [48]. Previous studies have reported increased levels of EDA-containing fibronectin together with reduced plasma fibronectin levels in patients with sepsis, vascular injury, and other severe inflammatory conditions [49–51]. Although contribution from plasma fibronectin cannot be excluded, the total fibronectin measured in lung tissue homogenates (Fig 2) likely reflects, at least in part, the EDA-containing cellular fibronectin. Thus, the reduction in tissue fibronectin following SPA4 peptide treatment may not only limit bacterial adherence but also attenuate TLR4-mediated inflammatory responses. Heat-inactivated SPA4 peptide was included as an additional control. Heat inactivation abolished the beneficial effects of SPA4 peptide on bacterial burden, edema, and inflammatory response [16], and similarly diminished its effects on fibrinogen, HMGB-1 and hyaluronan levels (Fig 2). In contrast, suppression of fibronectin was retained following treatment with the heat-inactivated SPA4 peptide. This observation suggests that peptide structural elements and motifs may have remained partially functional after heat treatment, although the mechanism underlying this effect remains unclear. Nonetheless, the fibronectin levels remained lower in infected mice treated with SPA4 peptide (17,925 ng/g lung) than in those treated with heat-inactivated SPA4 peptide (19,154 ng/g lung), indicating that the native peptide retains greater activity.

Unlike fibrinogen and fibronectin, HMGB-1 primarily functions as a released nuclear alarmin that amplifies inflammatory signaling through TLR4 and other receptors. HMGB-1 is a unique nuclear protein that undergoes modification (e.g., acetylation, methylation, phosphorylation, glycosylation, ubiquitination, and oxidation), translocated to cytoplasm, and released in the extracellular environment during infection and inflammation. Once released, extracellular HMGB-1 interacts with several cell surface receptors, including the receptor for advanced glycation end-products (RAGE), CXCR4, T cell immunoglobulin and mucin domain-3 (TIM-3), and TLRs, such as TLR4 [52,53]. Distinct structural domains of HMGB-1 mediate different biological functions. The A-box region (amino acids 9–79) exhibits anti-inflammatory activity, whereas the B-box region (amino acids 95–163) is primarily responsible for pro-inflammatory effects. In particular, amino acids 89–108 have been implicated in TLR4 binding and cytokine-inducing activity [54]. The biological activity of the extracellular HMGB-1 is further regulated by the redox state of its three cysteine residues (C23 and C45 within the A-box and C106 within the B-box). Fully reduced HMGB-1 acts as a chemoattractant, the disulfide bond linked HMGB-1 functions as a pro-inflammatory cytokine, and oxidation of these cysteines abolishes both activities [55].Although the present study did not evaluate specific HMGB-1 domains or redox isoforms, measurement of total HMGB-1 remains relevant given previous reports demonstrating elevated circulating HMGB-1 levels in patients with suspected community-acquired infections, pneumonia, and sepsis [56–58].

Hyaluronan, also known as hyaluronic acid, is a non-sulfated glycosaminoglycan present in a variety of tissues. In the lungs, hyaluronan is primarily localized to the peribronchial and interalveolar/perialveolar areas. It is a highly dynamic molecule whose biological activities are influenced by its molar mass, rate of synthesis, and accessibility to binding partners. High-molecular-weight hyaluronan species (>2,500 KDa) generally exhibit anti-inflammatory properties, whereas low-molecular-weight hyaluronan (<500 KDa) is associated with pro-inflammatory effects [59–61]. Despite these differential effects, increased tissue-expression of hyaluronan and its synthetic enzyme has been reported as early as 6 hours after LPS-induced inflammation and during active bacterial lung infection [62]. The ELISA kit used in this study detects both low- and high-molecular weight hyaluronan species (>35 KDa). Elevated hyaluronan levels, measured using similar ELISA-based assays, have also been reported in several other pulmonary conditions [4].

The limitation of this study is the evaluation of study parameters at a single post-infection time point (5 h). Per published studies [2–9], a single challenge with 1 x 10^7^ CFU of *P. aeruginosa* can induce rapid accumulation of significant amounts of PAMPs and DAMPs. However, detailed time-course studies are needed to characterize the temporal effects of SPA4 peptide on DAMP generation and trafficking. Such analyses will help determine whether the reduced DAMP levels are sustained after SPA4 peptide treatment. Based on the findings reported here, together with our previously published data demonstrating an improved lung pathology and function over 10 days of SPA4 peptide treatment in a *P. aeruginosa* LPS challenge model [20], we predict that single or multiple treatments with SPA4 peptide will maintain a reduced DAMP burden at later time-points. The coordinated activation of the “TLR4 radical cycle” or “damage chain reaction” by PAMPs and DAMPs is widely recognized as a key driver of chronic and persistent inflammatory disease [23–26]. Therefore, sustained suppression of DAMP accumulation may contribute to the long-term protective effects of SPA4 peptide. Nevertheless, the possibility that SPA4 peptide influences DAMP synthesis, degradation, cleavage, trafficking, distribution, or relative sizes of plasma and tissue pools due to edema, cannot be excluded and warrants further investigation. Regarding the results shown in Fig 3, although resident and infiltrating cells could not be distinguished, the reduced tissue cellularity is consistent with our previous findings of decreased inflammatory cell influx and tissue damage [16].

The development of peptide-based therapeutics has gained significant interest from both academic researchers and the pharmaceutical industry. Numerous peptides have been identified and investigated for their diverse biological activities and therapeutic potential. For example, amphibian-derived peptides such as RL-QN15, AL-VI10, OA-AL14, and FZ1 have been reported to modulate immune-epithelial crosstalk and inflammatory responses, while promoting hair follicle neogenesis, wound healing, and protection against oral cancer, vascular aging, and photoaging [63–66]. Human cathelicidin LL-37 has likewise been shown to regulate cytotoxicity, epithelial cell activation, angiogenesis, and wound repair [67]. Similarly, nesfatin-1, a peptide derived from human nucleobindin-2, exhibits antioxidative, anti-inflammatory, antiapoptotic, and antifibrotic effects in renal tissues, whereas catestatin, a chromogranin A-derived peptide, possesses chemotactic and antimicrobial properties [68,69] In this context, our research has focused on the human surfactant protein A (SP-A)-derived SPA4 peptide, particularly its pro-phagocytic and anti-inflammatory activities in models of infection and inflammation [16–22,70] This is the first time we have studied the TLR4 expression, DAMP levels, and total cellularity in lung tissues of *P. aeruginosa-*challenged, SPA4 peptide-treated mice. The findings provide additional evidence supporting the ability of SPA4 peptide to reduce bacterial burden, attenuate inflammatory responses, and mitigate lung injury.^16^ Furthermore, these results establish a foundation for future investigations using appropriate cellular systems, models of chronic infection and inflammation, genetic modifications, and pharmacological approaches to elucidate the molecular mechanisms through which SPA4 peptide regulates DAMPs and associated responses.

## Supporting information

S1 Raw Images File Raw blot images corresponding to the upper and lower panels shown in Fig 1B. (PDF)
