# Supplementary material for: SPA4 peptide attenuates the accumulation of selected damage-associated molecules and pulmonary cellularity during acute Pseudomonas aeruginosa infection in mice
Source: PLoS One. 2026 Nov 11;21(11):e0362301. doi: 10.1371/journal.pone.0362301 (PMC13754144; doi:10.1371/journal.pone.0362301)
Supplement: Supplementary file 1 [file pone.0362301.s001.pdf]

Raw blot image

Upper panel (Fig. 1B) TLR4

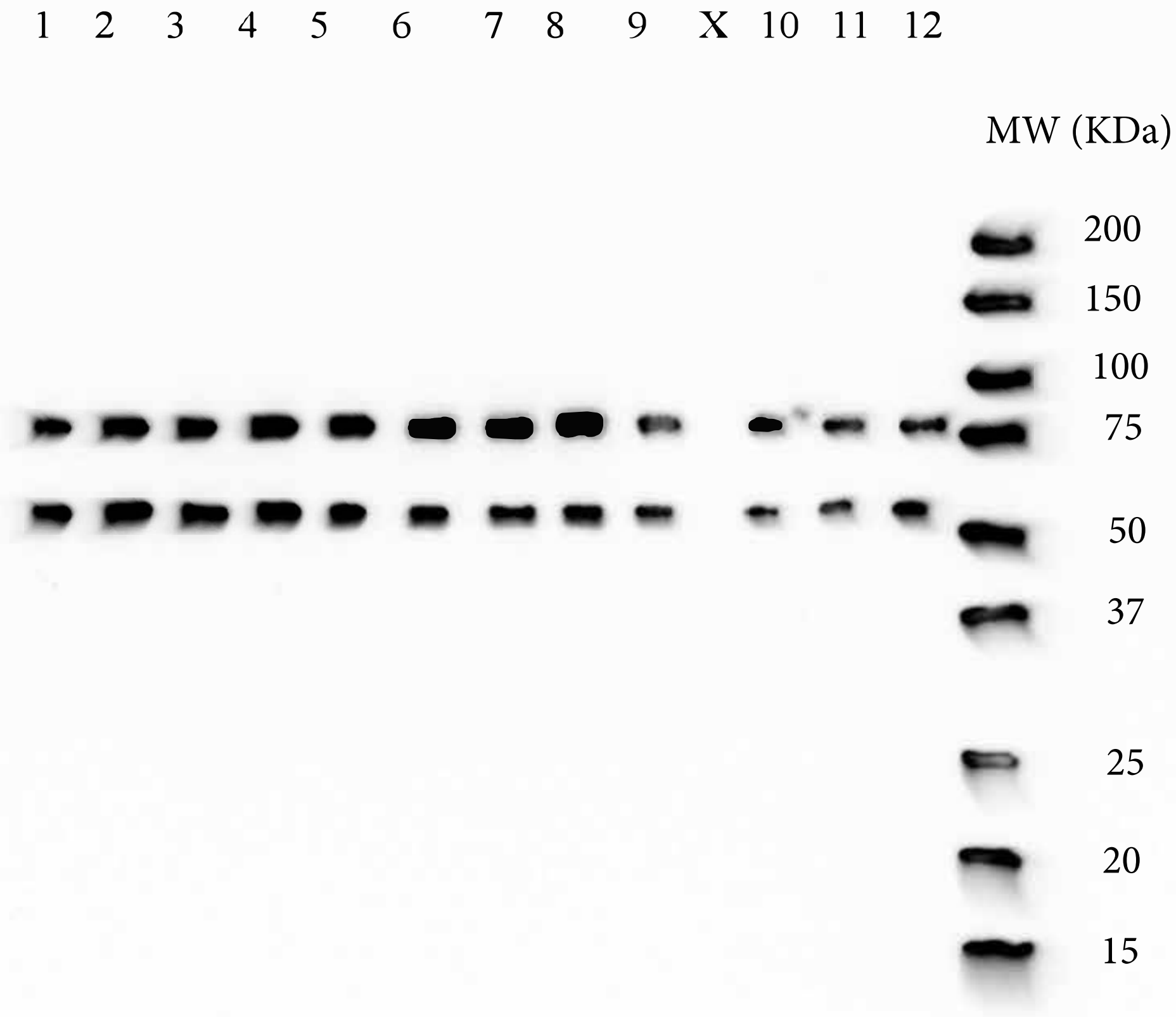

Lanes 1-3: *P. aeruginosa* + heat-inactivated SPA4 peptide, lanes 4-6: *P. aeruginosa* + vehicle, lanes 7-9: *P. aeruginosa* +SPA4 peptide, lane X: blank (no sample), lanes 10-12: Unchallenged, untreated

Raw blot image

Lower panel (Fig. 1B) β-actin

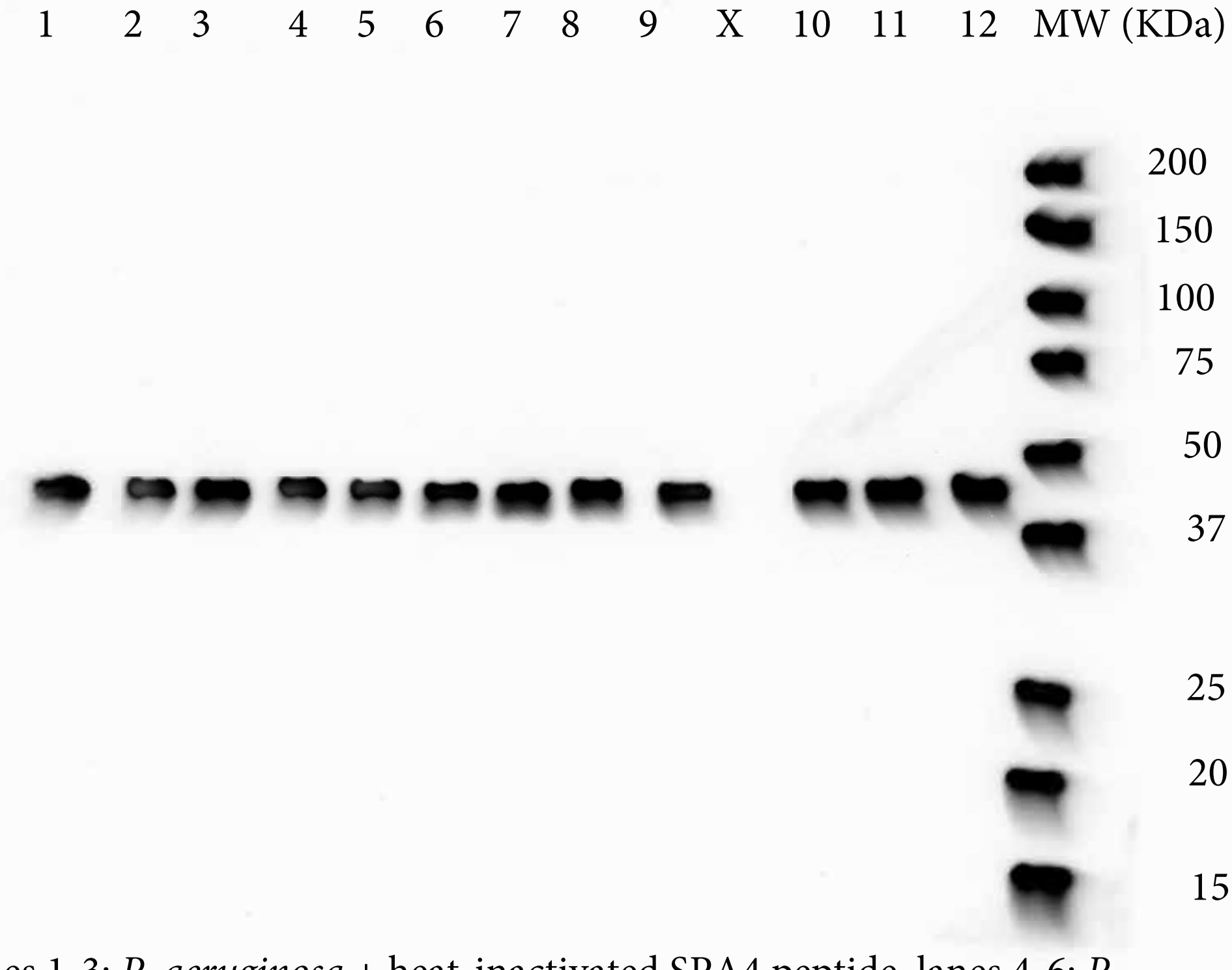

Lanes 1-3: *P. aeruginosa* + heat-inactivated SPA4 peptide, lanes 4-6: *P. aeruginosa* + vehicle, lanes 7-9: *P. aeruginosa* +SPA4 peptide, lane X: blank (no sample), lanes 10-12: Unchallenged, untreated
